# Single Intrastromal Voriconazole Injection for Refractory Aspergillus flavus Keratitis

**DOI:** 10.7759/cureus.116869

**Published:** 2026-09-24

**Authors:** Kaoutar Fahmaoui, Achraf Aneddame, Adil Mchachi, Laila Benhmidoune, Rachid Rayad

**Affiliations:** 1 Adult Ophthalmology Department, Faculty of Medicine and Pharmacy of Casablanca, 20 August 1953 Hospital, Ibn Rochd University Hospital Center, Hassan II University, Casablanca, MAR

**Keywords:** aspergillus flavus, deep stromal keratitis, fungal keratitis, intrastromal voriconazole, targeted drug delivery

## Abstract

Fungal keratitis is a potentially vision-threatening infection that can be difficult to treat when it involves the deep corneal layers. We report the case of a 50-year-old woman who developed deep central fungal keratitis following vegetative ocular trauma. Microbiological analysis identified *Aspergillus flavus *(*A. flavus*). Despite intensive topical treatment with voriconazole 1% and amphotericin B 0.15%, the infiltrate progressed after one week. A single intrastromal injection of voriconazole (50 µg/0.1 mL) was administered circumferentially around the infiltrate. Progressive clinical improvement followed, with complete resolution within one month and no recurrence during two years of follow-up. Final visual acuity was 20/100, with residual corneal scarring. This case highlights the potential role of intrastromal voriconazole as a targeted treatment option for deep, refractory fungal keratitis.

## Introduction

Fungal keratitis is a significant cause of visual impairment. Its high prevalence in developing countries is associated with delayed presentation and limited access to appropriate treatment. It accounts for nearly half of infectious keratitis cases in these settings and generally has a worse prognosis than bacterial keratitis [1-3]. Risk factors include ocular trauma with vegetative matter, contaminated contact lenses, pre-existing ocular surface disorders, and inappropriate use of topical corticosteroids [4,5].

Treatment remains challenging because most topical antifungals have limited penetration into the deeper corneal layers and may cause ocular surface toxicity [6]. When inadequately treated, fungal keratitis can result in corneal melting, perforation, endophthalmitis, and the need for therapeutic keratoplasty [4,7]. Intrastromal injection of antifungal agents such as voriconazole may overcome the limitations of topical therapy by achieving higher drug concentrations at the site of infection and has shown encouraging results in deep, refractory fungal keratitis [8-10]. However, the optimal dose, number and timing of injections, and pathogen-specific response are not yet well established [10].

*Aspergillus* species are important causes of filamentous fungal keratitis, with *Aspergillus flavus *(*A. flavus*) reported as a frequent etiologic species [1]. Although many *Aspergillus* infections respond to medical therapy, deep corneal lesions may be more difficult to treat and may require surgical intervention [1,5]. We report a case of deep *A. flavus* keratitis that progressed despite intensive topical antifungal therapy and resolved following a single intrastromal voriconazole (ISV) injection combined with continued topical antifungal therapy.

## Case presentation

A 50-year-old woman presented with pain, redness, and photophobia for five days. She lived in a rural area and reported a history of vegetative ocular trauma. She denied using any topical drops or traditional ocular remedies before presentation. Best-corrected visual acuity was limited to hand motion. Slit-lamp examination revealed conjunctival congestion and a 6 mm central, dense, grayish mid-stromal infiltrate with surrounding stromal edema and no anterior chamber reaction. Intraocular pressure was 12 mmHg. Fundus examination was unremarkable to the extent visualized. Examination of the left eye was unremarkable.

Given the history of vegetative ocular trauma and the presence of a deep stromal infiltrate with irregular margins, fungal keratitis was considered more likely than bacterial keratitis. However, bacterial keratitis could not be excluded based on the clinical findings alone, and corneal scraping was therefore performed for microbiological evaluation. Empirical treatment with topical voriconazole 1%, amphotericin B 0.15%, ceftazidime 5%, and vancomycin 2.5% was initiated immediately, with all agents administered hourly. Preservative-free lubricants were started after two days to reduce ocular surface toxicity.

Direct microscopy revealed fungal hyphae, and fungal culture on Sabouraud dextrose agar yielded *A. flavus* after 48 hours, identified by its characteristic macroscopic and microscopic morphology. Antifungal susceptibility testing was not performed. All bacterial cultures remained sterile, with no bacterial growth detected. Based on the microbiological results, the fortified antibiotics were discontinued, and antifungal therapy was continued. Despite one week of intensive medical treatment, the corneal infiltrate increased in size from 6 to 6.5 mm (Figure 1).

**Figure 1:**
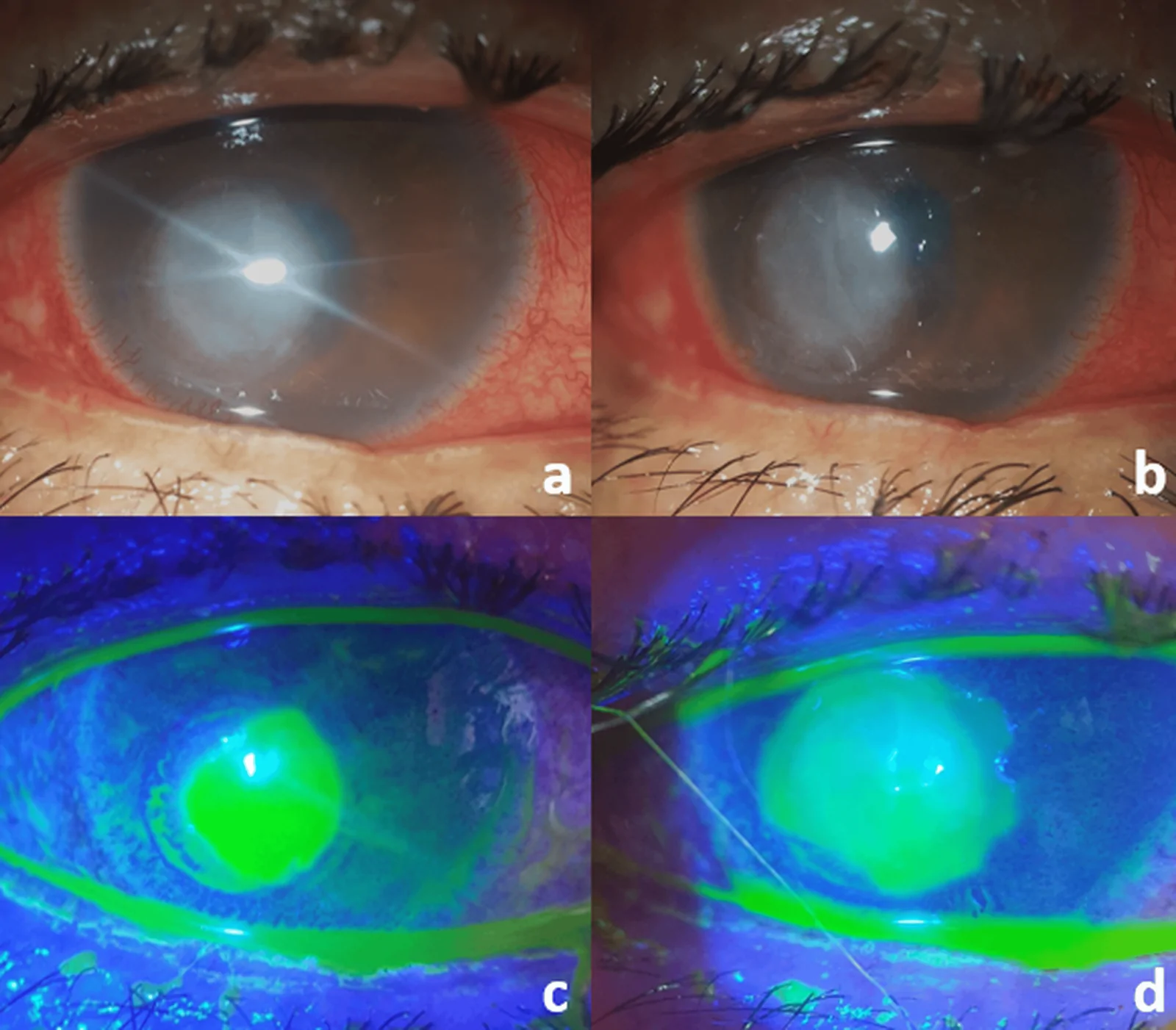
Progression of deep fungal keratitis despite intensive topical antifungal therapy (a, c) At presentation, showing a dense, central, approximately 6 mm grayish mid-stromal infiltrate with surrounding stromal edema. (b, d) After one week of hourly topical voriconazole 1% and amphotericin B 0.15%, showing enlargement of the central infiltrate and persistent stromal edema

Given the progressive and refractory nature of the keratitis, a single ISV injection was subsequently performed. Voriconazole (200 mg) was reconstituted and diluted to a final concentration of 0.5 mg/mL (50 µg/0.1 mL). Under local anesthesia and strict aseptic conditions, a 30 gauge needle was used to administer five perilesional mid-stromal deposits, creating a circumferential barrier around the infiltrate (Figure 2).

**Figure 2:**
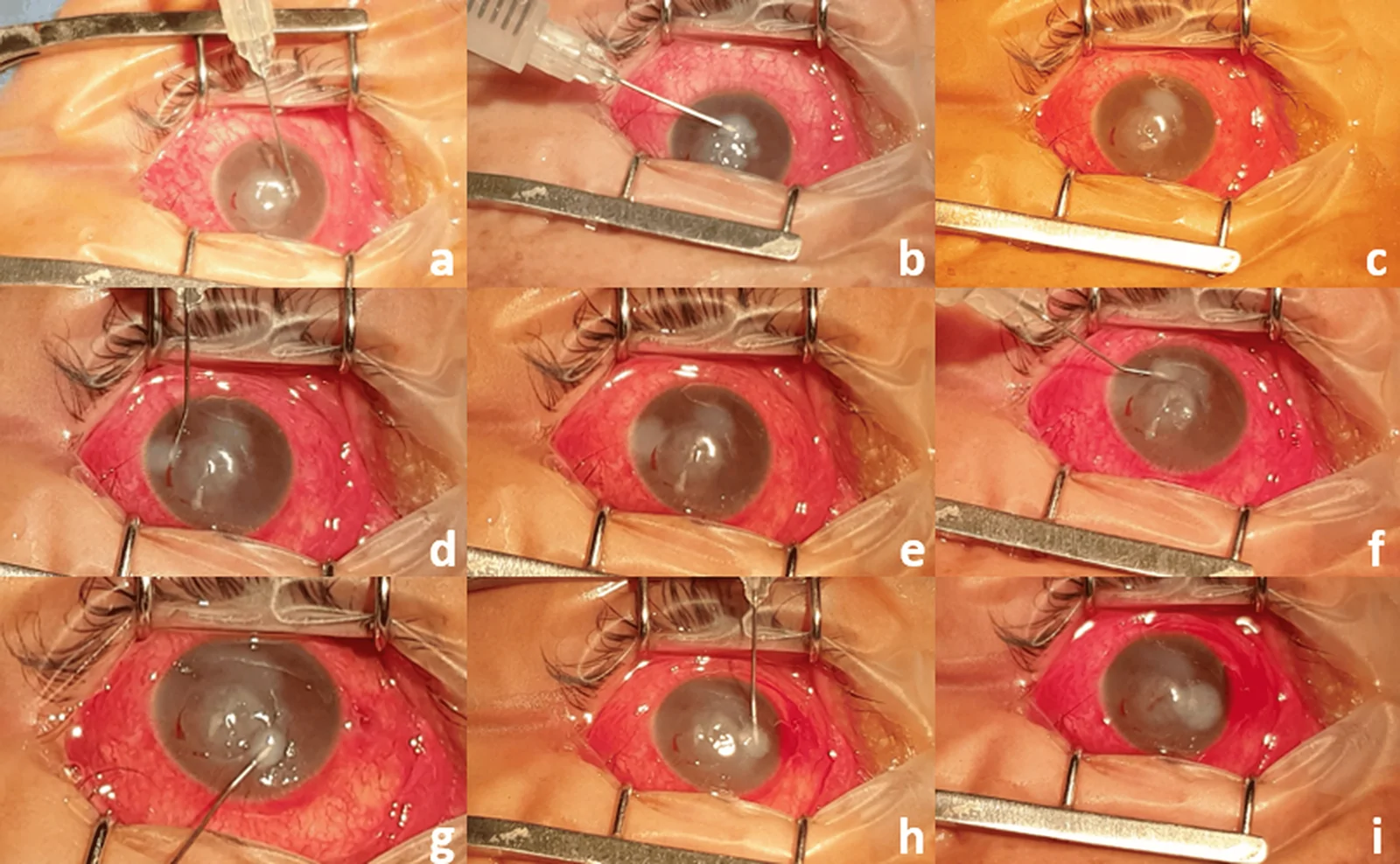
Intrastromal voriconazole injection (a-i) Sequential photographs showing the administration of five perilesional mid-stromal deposits of voriconazole using a 30 gauge needle, placed circumferentially around the infiltrate to create a barrier around the infected area

No procedure-related complications or adverse events were observed during or after the ISV, and no signs of corneal thinning or perforation were noted during follow-up. Clinical improvement was first noted two days after the injection, with reduced pain, stromal edema, and infiltrate size (5 mm versus 6 mm at baseline). At this time, topical voriconazole 1% and amphotericin B 0.15% were each tapered to eight drops per day. By day 15, further improvement was observed, with regression of symptoms and conjunctival congestion and further reduction of the infiltrate to 3 mm. By day 30, the infiltrate had completely resolved, leaving residual corneal stromal scarring (Figure 3).

**Figure 3:**
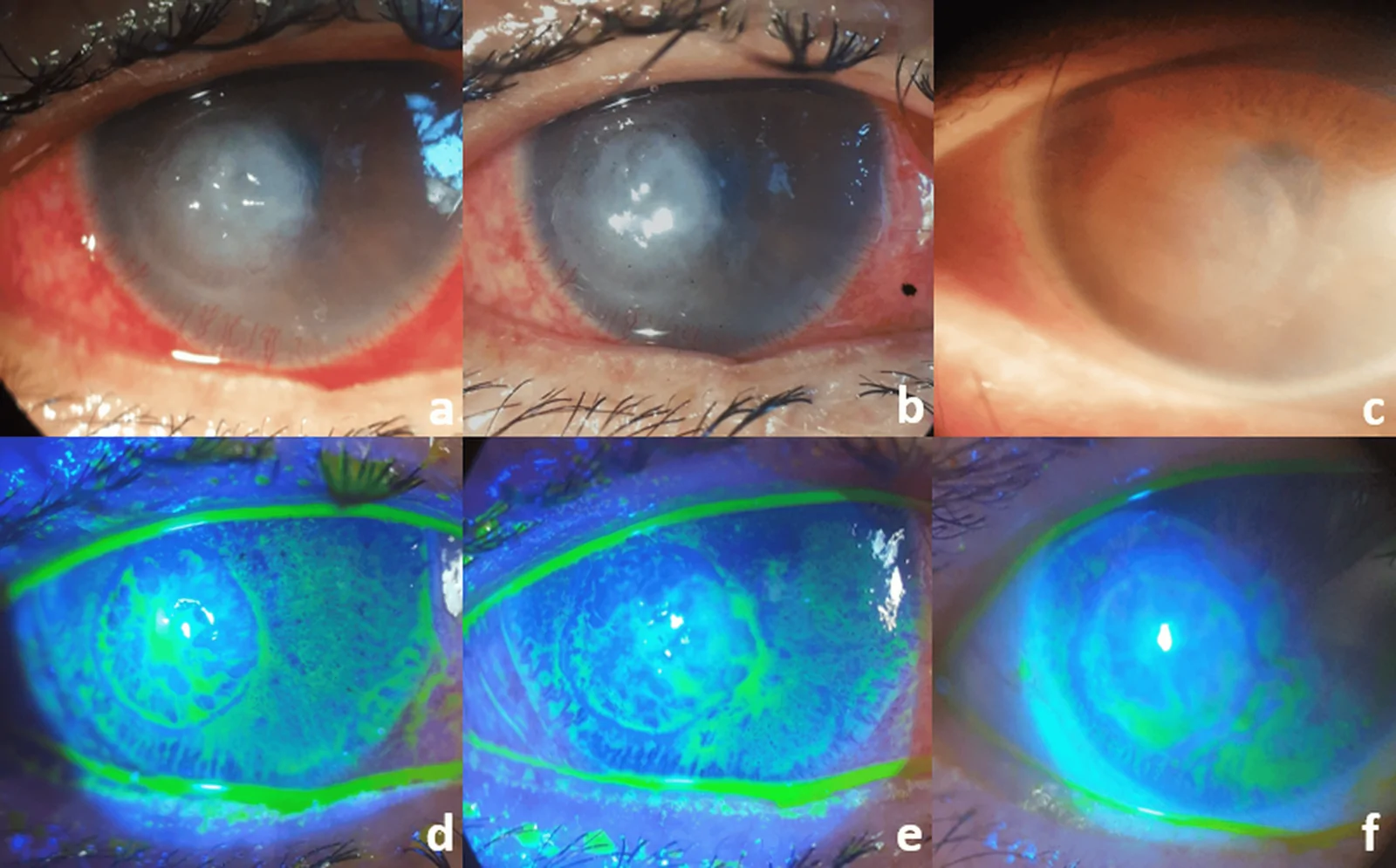
Clinical response following a single intrastromal voriconazole injection (a, d) Two days after injection, showing early reduction of the stromal infiltrate. (b, e) Fifteen days after injection, showing further reduction in infiltrate size and improvement of stromal edema. (c, f) Thirty days after injection, showing complete resolution of the infiltrate with residual stromal scarring

Topical voriconazole 1% and amphotericin B 0.15% were then each tapered to four drops per day and continued for an additional month before being discontinued. There was no evidence of recurrent infection during two years of follow-up, and visual acuity improved to 20/100.

## Discussion

Deep stromal fungal keratitis remains challenging because topical antifungal agents may not achieve adequate concentrations at the site of infection. Voriconazole is a broad-spectrum triazole with good in vitro activity against several clinically important filamentous fungi, including species of the genus *Aspergillus*, and intrastromal administration may overcome the limitations of topical therapy by delivering high drug concentrations directly to the infected stroma [4,11].

Several studies have reported encouraging results with ISV at a concentration of 50 µg/0.1 mL, typically injected circumferentially around the infiltrate [3,8,9,12]. In a series of 25 culture-proven cases, resolution was achieved in 72% of eyes, although repeated injections were sometimes required and infections caused by *Fusarium* species appeared less responsive [12]. More recently, complete corneal healing was reported in a series of 21 patients, with one to three injections administered according to clinical response [9].

Our case demonstrated a favorable response following a single ISV injection in culture-proven deep *A. flavus* keratitis. This is noteworthy because filamentous fungal keratitis, including infections caused by *Aspergillus* and *Fusarium* species, may remain refractory despite intrastromal therapy, and repeated injections are often required [4,12]. The clinical and microbiological characteristics that may predict successful treatment with a single ISV injection remain uncertain. In the present case, the relatively localized infiltrate, culture-proven *A. flavus* infection, and/or the prompt addition of targeted intrastromal therapy may have contributed to the outcome; however, these observations cannot establish predictors of response.

The clinical response should also be interpreted in the context of the concomitant topical antifungal therapy. Because topical treatment was continued after ISV, the complete resolution of the infection cannot be attributed to ISV alone. Nevertheless, the patient's improvement followed the addition of intrastromal voriconazole to the ongoing topical treatment. ISV should therefore be considered an adjunct to, rather than a replacement for, appropriate topical antifungal therapy.

Optimal dosing and injection intervals remain uncertain, and pathogen-specific efficacy has not been established. Our report is limited by its single-case design and the absence of antifungal susceptibility testing, which prevents assessment of the organism's in vitro susceptibility to voriconazole. Nevertheless, the complete clinical resolution and absence of recurrence over two years provide clinical support for the potential role of ISV as an adjunctive treatment in selected cases of deep *A. flavus* keratitis. Further studies are needed to clarify patient selection, optimal dosing, injection intervals, and pathogen-specific outcomes.

## Conclusions

ISV may be a useful targeted adjunctive treatment for deep fungal keratitis that is refractory or responds inadequately to topical therapy. In this case of culture-proven *A. flavus* keratitis, progressive clinical improvement with complete resolution of the infiltrate by one month was observed following a single ISV injection combined with continued topical antifungal therapy. Although the favorable outcome cannot be attributed to ISV alone, this case illustrates its potential role in selected cases of deep fungal keratitis.
